# Independent component analysis of Alzheimer's DNA microarray gene expression data

**DOI:** 10.1186/1750-1326-4-5

**Published:** 2009-01-28

**Authors:** Wei Kong, Xiaoyang Mou, Qingzhong Liu, Zhongxue Chen, Charles R Vanderburg, Jack T Rogers, Xudong Huang

**Affiliations:** 1 Information Engineering College, Shanghai Maritime University, Shanghai 200135, PR China; 2 Biomedical Informatics and Cheminformatics Group, Conjugate and Medicinal Chemistry Laboratory, Division of Nuclear Medicine and Molecular Imaging and Center for Advanced Medical Imaging, Department of Radiology, Brigham and Women's Hospital and Harvard Medical School, Boston, MA 02115, USA; 3 Computer Science Department and Institute for Complex Additive Systems Analysis, New Mexico Institute of Mining and Technology, Socorro, NM 87801, USA; 4 Joseph Stokes Jr. Research Institute, Children's Hospital of Philadelphia, Philadelphia, PA 19104, USA; 5 Advanced Tissue Resource Center, Harvard NeuroDiscovery Center and Department of Neurology, Massachusetts General Hospital and Harvard Medical School, Charlestown, MA 02129, USA; 6 Neurochemistry Laboratory, Department of Psychiatry, Massachusetts General Hospital and Harvard Medical School, Charlestown, MA 02129, USA

## Abstract

**Background:**

Gene microarray technology is an effective tool to investigate the simultaneous activity of multiple cellular pathways from hundreds to thousands of genes. However, because data in the colossal amounts generated by DNA microarray technology are usually complex, noisy, high-dimensional, and often hindered by low statistical power, their exploitation is difficult. To overcome these problems, two kinds of unsupervised analysis methods for microarray data: principal component analysis (PCA) and independent component analysis (ICA) have been developed to accomplish the task. PCA projects the data into a new space spanned by the principal components that are mutually orthonormal to each other. The constraint of mutual orthogonality and second-order statistics technique within PCA algorithms, however, may not be applied to the biological systems studied. Extracting and characterizing the most informative features of the biological signals, however, require higher-order statistics.

**Results:**

ICA is one of the unsupervised algorithms that can extract higher-order statistical structures from data and has been applied to DNA microarray gene expression data analysis. We performed FastICA method on DNA microarray gene expression data from Alzheimer's disease (AD) hippocampal tissue samples and consequential gene clustering. Experimental results showed that the ICA method can improve the clustering results of AD samples and identify significant genes. More than 50 significant genes with high expression levels in severe AD were extracted, representing immunity-related protein, metal-related protein, membrane protein, lipoprotein, neuropeptide, cytoskeleton protein, cellular binding protein, and ribosomal protein. Within the aforementioned categories, our method also found 37 significant genes with low expression levels. Moreover, it is worth noting that some oncogenes and phosphorylation-related proteins are expressed in low levels. In comparison to the PCA and support vector machine recursive feature elimination (SVM-RFE) methods, which are widely used in microarray data analysis, ICA can identify more AD-related genes. Furthermore, we have validated and identified many genes that are associated with AD pathogenesis.

**Conclusion:**

We demonstrated that ICA exploits higher-order statistics to identify gene expression profiles as linear combinations of elementary expression patterns that lead to the construction of potential AD-related pathogenic pathways. Our computing results also validated that the ICA model outperformed PCA and the SVM-RFE method. This report shows that ICA as a microarray data analysis tool can help us to elucidate the molecular taxonomy of AD and other multifactorial and polygenic complex diseases.

## Background

Since microarray technology can determine the expression levels of thousands of genes from a single array of chemical sensors, it has become a popular gene expression screening tool in the molecular investigation of various diseases. This technology allows for two main types of descriptive analyses: firstly, the identification of genes that may be responsible for a clinicopathological feature or phenotype, and secondly, the genomic classification of tissue.

Its ultimate goal is to improve clinical outcome by adapting therapy based on the molecular characteristics of human diseases such as a tumor [1,2]. Various methods have been developed to accomplish these tasks. However, most methods only consider individual genes, making the results difficult for biologists to interpret due to the large number of genes, their complex underlying inter-gene dependency, and the high co-linearity among the gene expression profiles.

Therefore, to understand the coordinated effects of multiple genes, researchers need to extract the underlying features from the multi-variable dataset and thereby reduce dimensionality and redundancy inherent in the measured data. To extract these features, however, any microarray technology, to be truly effective, must address the issue of noise in the array systems that lead to imperfection in experimental design. Additionally, to discover functional modules involved in gene regulatory or signaling pathways, powerful mathematical and computational methods are needed for modeling and analyzing the microarray data of interest.

Two kinds of unsupervised analysis methods for microarray data analysis, principal component analysis (PCA) and independent component analysis (ICA), have been developed to accomplish the tasks. PCA projects the data into a new space spanned by the principal components. Each successive principal component is selected to be orthonormal to the previous ones and to capture the maximum information that is not already present in the previous components. The constraint of mutual orthogonality of components implied in classical PCA methods, however, may not be suitable for biological systems. Biological model components are usually statistically independent and without the constraint of orthogonality. Hence, ICA is well suited to biological data because it assumes that the gene expression data generated from the DNA microarray technology is a linear combination of some independent components having specific biological interpretations. Another useful advantage of ICA is that it does not use any training data and *a priori *knowledge about a parameter of its data filtering and mixing.

Hori in 2001 [3,4] and Liebermeister in 2002 [5] showed that the ICA model can effectively classify gene expressions into biologically meaningful groups and relate them to distinct biological processes. Thus ICA has been widely used in DNA microarray data analysis for feature extraction, clustering, and the classification of gene regulation analysis. Most published literature on the use of ICA analysis for microarray data are about yeast cells' cycle [6-8] and cancer data such as: ovarian cancer [9], breast cancer [10-13], endometrial cancer [14], colon and prostate cancer [15,16], and acute myeloid leukemia [17], etc.

Although the exact causes of AD are not fully revealed, DNA microarray technique has been applied to AD-related gene profiling. However, in our knowledge, application of ICA in AD-related DNA microarray data analysis has not been reported before. Since ICA can both identify gene expression patterns and group genes into expression classes that might provide much greater insight into biological function and relevance, we employed ICA methods to uncover biologically meaningful patterns in AD microarray gene expression data. Herein, we present a new computational approach to reveal AD-related molecular taxonomy and to identify AD pathogenesis-related genes.

## Results

To perform ICA application in AD gene expression data analysis, we used a dataset from GEO DataSets deposited by Blalock et al that featured hippocampal gene expression from control and AD samples [18]. The hippocampal specimens were obtained through the Brain Bank of the Alzheimer's Disease Research Center at the University of Kentucky. The human GeneChips (HG-U133A) of Affymetrix and Microarray Suite 5 were used in the microarray data collection. The procedures for total RNA isolation, labeling, and microarray were described in [18] and [19].

We excluded the samples with significant noise and chose 8 control and 5 severe AD samples for ICA application, with each sample containing 22283 gene expressions. In addition, since microarray data often yield "unregulated" genes, whose expression profile does not contain much information, we filtered out unregulated genes prior to applying ICA. Finally, to perform ICA, we selected 13 samples (8 control and 5 severe AD samples) and 3617 genes from each sample.

### ICA Decomposed AD Microarray Data into Biological Processes

The main modeling hypothesis underlying the application of ICA to gene expression data analysis is that the gene expression level is determined by a linear combination of biological processes, many of which may up-regulate or down-regulate gene expression. It is assumed that these biological processes correspond to activation or inhibition of single pathways or a network of highly correlated pathways, and that each of these pathways only affects a relatively small percentage of all genes. Because of the statistical independence assumption inherent in the ICA inference process, we would expect ICA components to map closer to pathways.

**Figure 1:**
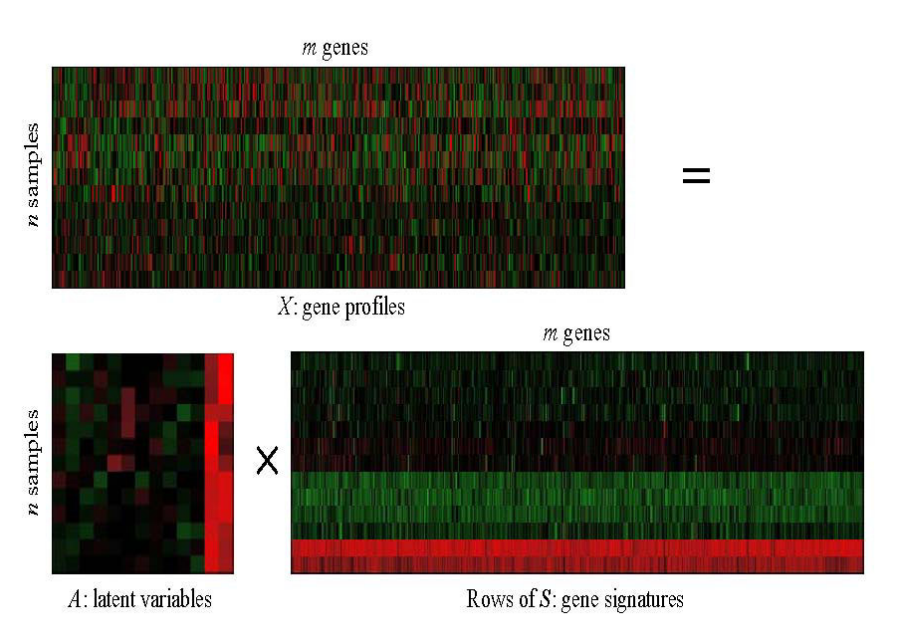
**ICA decomposition results of AD microarray gene expression data**. FastICA was applied to the AD microarray data matrix *X *with 13 samples and 3617 genes. Using the ICA method, *X *= *AS*, FastICA algorithm decomposes matrix *X *(13 × 3617) into latent variable matrix *A *(13 × 13) and gene signature matrix *S *(13 × 3617).

Figure 1 gives the ICA decomposition results of the 13 × 3617 AD microarray data matrix X. Each observed sample was considered a linear combination of gene signatures captured by ICA under the weights of rows of matrix ***A***. Here, FastICA, presented by Hyvärinen [20], was applied to the AD microarray data matrix ***X ***with rows corresponding to *n *(13) samples and columns corresponding to *m *(3617) genes. It decomposes matrix ***X ***into latent variable matrix ***A ***(13 × 13) and gene signature matrix ***S ***(13 × 3617). In FastICA algorithm, nonlinear function *g*(*u*) = *tanh*(*a*1*u) was used as the probability density distribution of the outputs *u *during the iteration, where here *a*1 is a constant. As the FastICA algorithm relies on random initializations for its maximization and faces the problem of convergence to local optima, Chiappetta et al. [10] presented consensus components by rerunning the FastICA algorithm with random initializations and Himberg et al. [21] proposed resampling of ICA components and used estimated centrotypes as the representatives of ICA components. In our study, we iterated FastICA 50 times to alleviate the instability of the slightly different results generated from each iteration. The final components were estimated as the centrotypes of the iterated estimates for each component.

### ICA Improved Gene Clustering Results of AD Samples

#### Sample clustering by matrix A

ICA essentially seeks a new representation of the observed expression profile matrix ***X ***with the columns of matrix ***A ***representing the new basis vectors (latent variables). Each row of ***A ***contains the weight with which gene signatures contribute to observed expression profiles.

**Figure 2:**
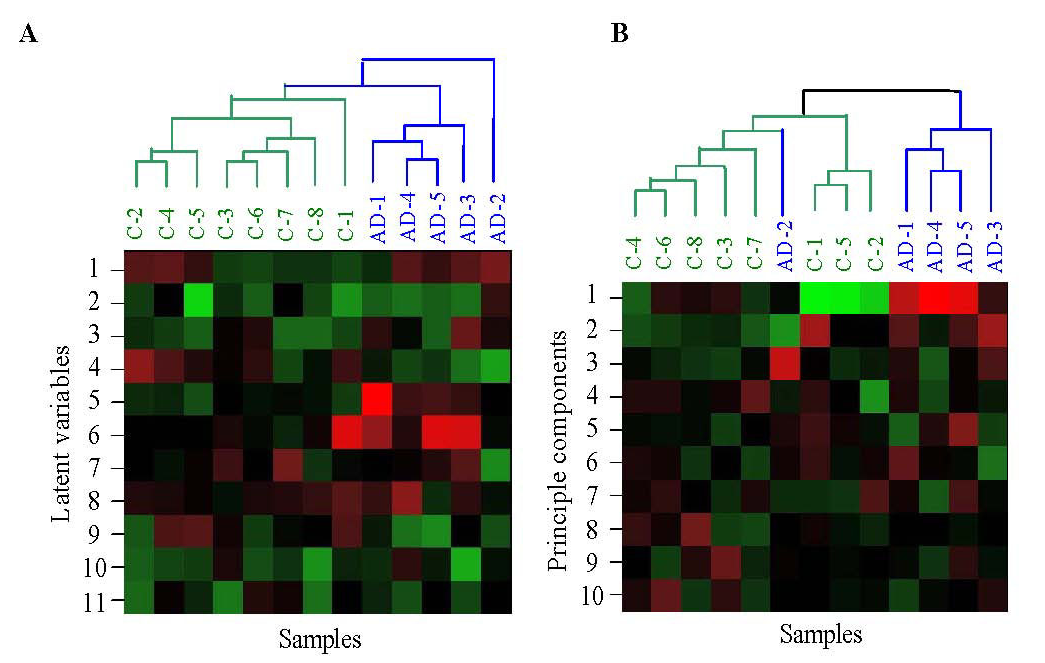
**Hierarchical clustering of the ICA and PCA outputs**. **(A) **Hierarchical clustering of the ICA outputs with the last two 'common' components of matrix *A *removed. To display the cluster dendrogram conveniently, we transposed matrix *A *in the graph. That is, the columns of the graphical representation correspond to the rows of matrix *A*, denoting samples, and the rows of the graphical representation correspond to the columns of matrix *A*, denoting the ICA latent variables. **(B) **Hierarchical clustering of the principle components, with the number of the principle components *k *= 10. Similarly, the rows and columns of the graph denote the principle component and samples, respectively.

Unsupervised hierarchical clustering was applied to rows of ***A ***to validate the efficiency of ICA outputs (Figure 2A). The clustering method was performed on rows of matrix ***A ***with the last two latent variables removed that were considered noise or, since their values are similar across all samples, to have no biological relevance to AD. See the graphical representation of matrix ***A ***in Figure 1. As can be seen from the clustering dendrogram, the first 11 ICA latent variables captured sufficient biologically significant information from samples data. The control and severe AD samples can be clearly discriminated. Similar hierarchical clustering was done on the principal components (PCs) outputted by PCA on the same original data (where the gene expression measurements are the variables and the samples are the observations) in Figure 2B. In the PCA method, the observed samples were represented as a linear combination of the PCs with associated gene scores. Here, the first 10 PCs capturing 95.5% variance were selected to represent the original data, and the remaining PCs with lower variance that contained noise were removed for the clustering. Figure 2B showed that it can cluster some of the control samples and severe AD samples, however, the severe AD sample 'AD-2' cannot be discriminated from control samples.

#### Sample clustering by reconstructed data

**Figure 3:**
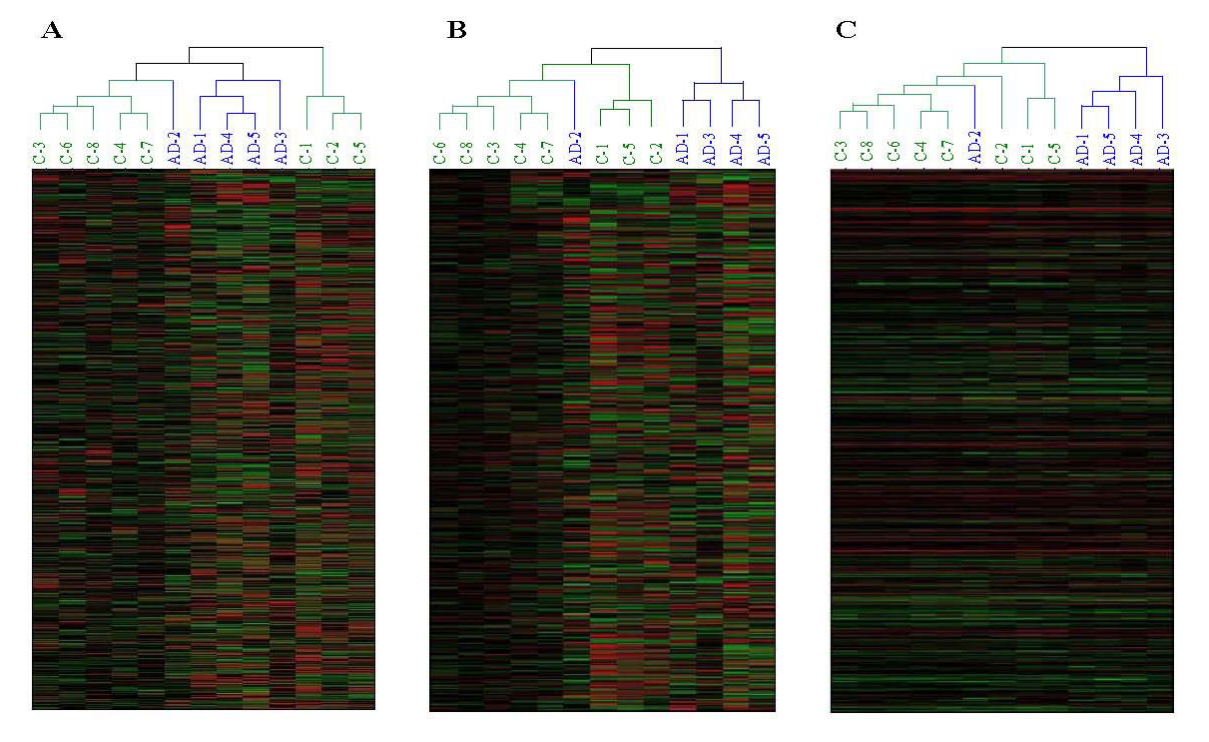
**Unsupervised hierarchical clustering of the normalized raw data**. **(A) **Data reconstructed by PCA; **(B) **and the data reconstructed by FastICA; **(C) **C1–8: control samples, AD1–5: severe AD samples. Red and green blocks represent signal increase and decrease from the mean respectively. For the PCA reconstructed data, the first 10 principle components were applied and their cumulative contribution of the corresponding eigenvalues was 95.5%. For ICA-derived data, the genes with loadings that exceed the threshold (= 2) were considered significant, and the remaining genes with lower values were considered as noises and set to zero. Here, by comparing them to the original data, both PCA and ICA-derived data greatly improved the clustering results of AD microarray data.

ICA transfer is an adaptive process in which the independent components are as sparse as possible. Lee et al. (18) demonstrated that the underlying biological processes are more super-Gaussian than original inputs microarray gene data. For this property, one may assume that the activities of components with small absolute values are noisy or have redundancy information and must be set to zero, retaining just a few components with large activities, ***S ***→ ***S***_*new*_. Then the newly reconstructed data are obtained by ***X***_*new *_= ***A S***_*new*_. Figure 3 shows the unsupervised hierarchical clustering was applied to the normalized raw data, with the data reconstructed by PCA and FastICA, respectively.

For the original data (Figure 3A), some of the control samples and severe AD samples have been clustered together, but the highest hierarchical split did not separate the two classes as would have been expected. For the data reconstructed by both PCA and ICA, the clustering results were greatly improved (Figure 3B and 3C). In PCA method, the first 10 components associated with a larger variance were selected to reconstruct data that captured most of the information (the cumulative contribution of their eigenvalues exceeded 95.5%) of original data whereas the remaining components with lower variance contained noise and were removed. The ICA method extracted *m *(13) gene signatures (rows of matrix ***S***) that were mutually statistically independent as underlying biological processes. Each independent component was as sparse as possible, in which only a few relevant genes were significantly affected, leaving the majority of genes relatively unaffected. The filtering capacity of ICA was achieved by setting the entries in each gene signature with values that are less than the threshold = 0. Then, the reconstructed data gave a clearer clustering result to discriminate control and severe AD samples from original data.

**Figure 4:**
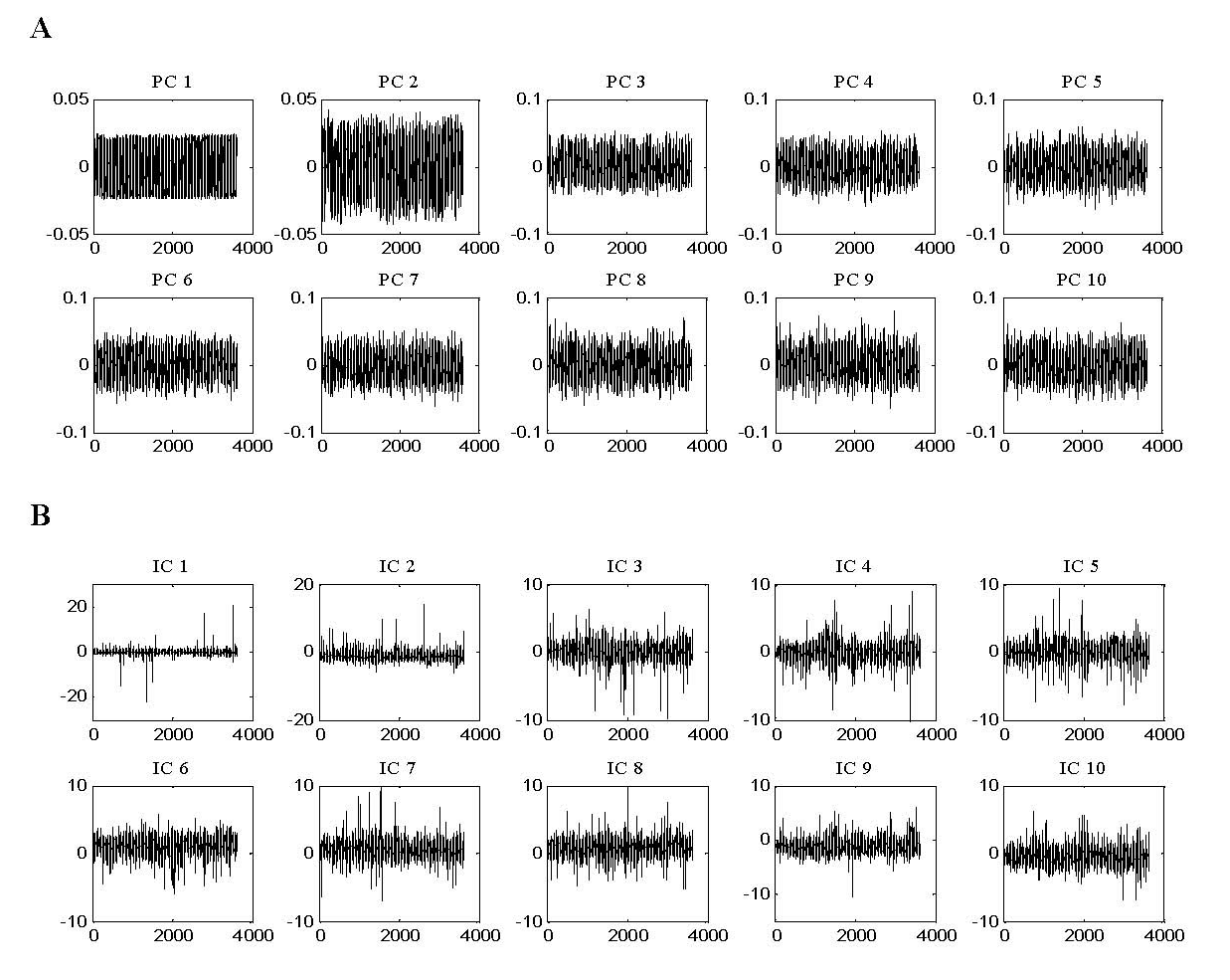
**The first 10 PCs and ICs extracted by PCA and ICA methods respectively**. **(A) **The first 10 PCs obtained by PCA that capture 95.5% information of original AD microarray data. **(B) **The first 10 ICs (gene signatures) uncovered by ICA from AD microarray data in which only a few relevant genes were significantly affected, leaving the majority of genes unaffected. The x-axis in each graph denotes genes, and the y-axis represents relative signal intensity.

**Figure 5:**
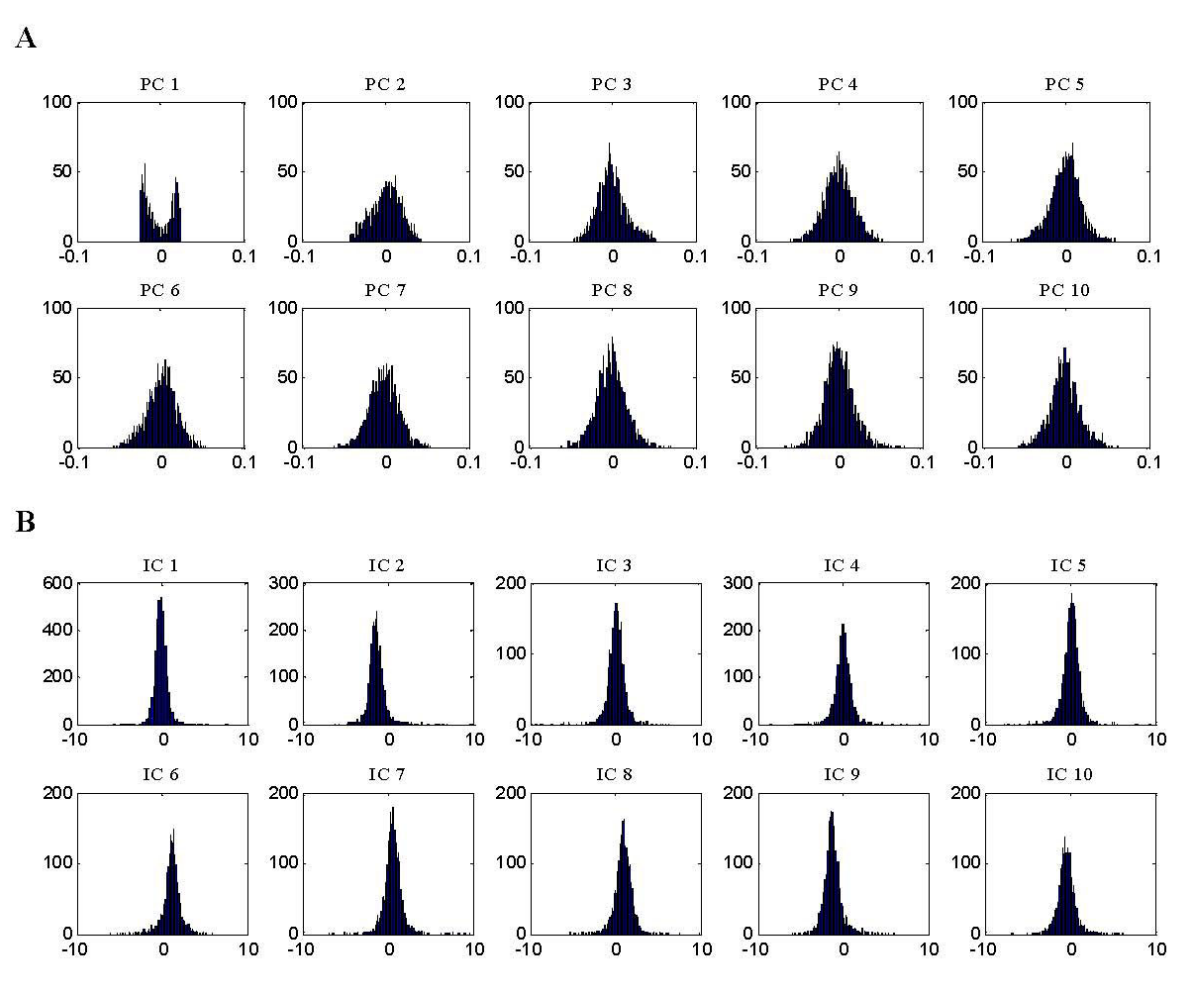
**The corresponding histograms of the first 10 PCs (A) and ICs (B) in figure 4**. The histograms of ICs in (B) displayed more super-Gaussian than did that of the PCs (A). ICA extracted sparser gene signatures, and, since each of gene signature only affects a relatively small percentage of all genes, we can expect that ICA found more significant genes related to AD.

Although ICA produced similar results to PCA on AD sample clustering, it extracted sparser gene signatures that, since each gene signature only affects a relatively small percentage of all genes, were more useful for finding significant genes related to AD. Figure 4 showed the graphical representation of gene signatures unveiled by PCA (A) and ICA (B). We would expect the identified ICs to map more closely to known pathways than PCA that does not use the statistical independence criterion (we will discuss the discovery of co-regulated genes associated with AD in the next section). Figure 5 showed the histogram of the corresponding PCs and ICs. The underlying biological processes extracted by ICA are more super-Gaussian (sparser) than the PCs.

### ICA Identified Significant Genes for AD

**Figure 6:**
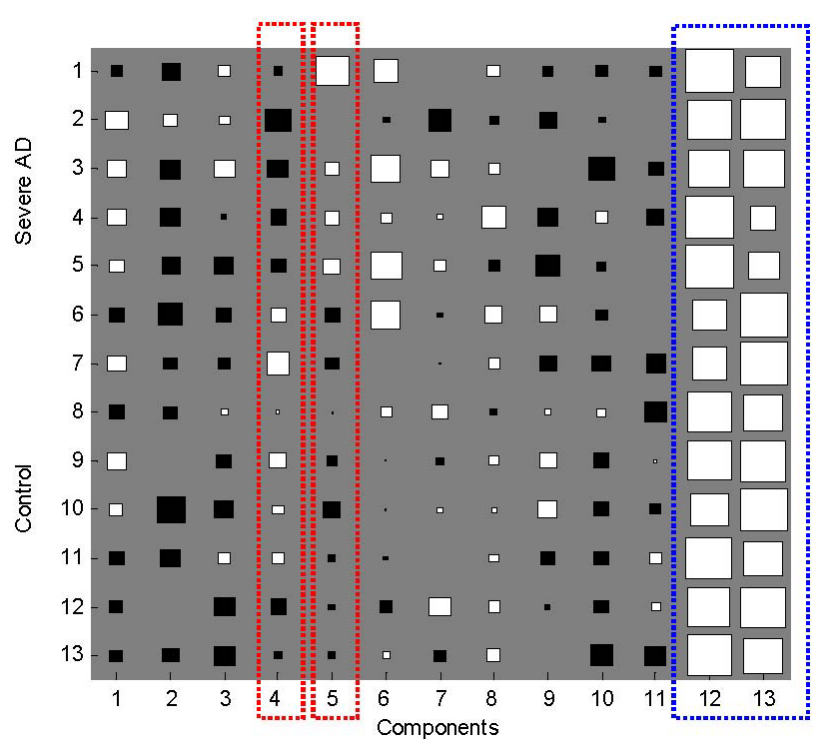
**Hinton diagram representation of latent variable matrix *A***. The size of each square corresponds to the amount *a*_*nm *_of component *m *in sample *n*. White and black represent positive and negative values, respectively.

In the ICA outputs matrix, each row of matrix ***A ***contained the weights with which the expression levels of the *m *genes contribute to the corresponding observed expression profile (row of matrix ***X***). Therefore, the profile order of rows of ***A ***is the same as that of the observed expression profiles, and each column of ***A ***is associated with the corresponding gene signature (IC). Figure 6 shows the Hinton diagram representation of matrix ***A ***derived by FastICA.

**Figure 7:**
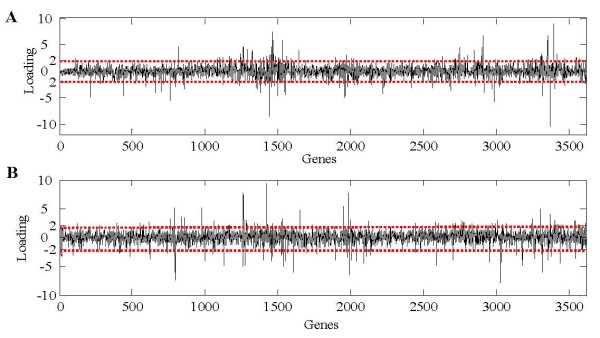
**The 4-th (A) and 5-th (B) gene signatures (corresponding to the 4-th and 5-th column of matrix *A *in figure 6)**. Genes with loadings that exceed the chosen threshold (red line) were considered significant Here, the threshold = 2. The positive and negative loadings correspond to up- and down-regulation of expression, respectively.

The original data set consisted of 5 severe AD microarray gene expressions (first 5 rows) and 8 control samples (last 8 rows), so this assignment is also valid for the rows of ***A***. In Figure 6, since the sign of the values are distinctly different, the 4-th and 5-th columns of ***A ***(red circles) discriminate between severe AD and the control samples. For example, the values of the 4-th latent variable (4-th columns of ***A***) are all negative for severe AD samples (first 5 rows) and positive for most of the control samples (last 8 rows) and the values of the 5-th latent variable (5-th columns of ***A***) are all positive for severe AD samples (first 5 rows) and positive for all the control samples (last 8 rows). So the identified latent variables can be related to their corresponding gene signatures (the 4-th and 5-th row of matrix ***S***) to find those individual genes that strongly contribute to that component. In addition, the last two columns of matrix ***A ***seem to have no biological relevance to AD because their values are similar across all the samples (blue circle). These 'common' components can be ignored from the later investigation. Figure 7 shows the corresponding gene signatures in matrix ***S ***(4-th and 5-th row of ***S***) for the 4-th and 5-th component in matrix ***A***.

**Figure 8:**
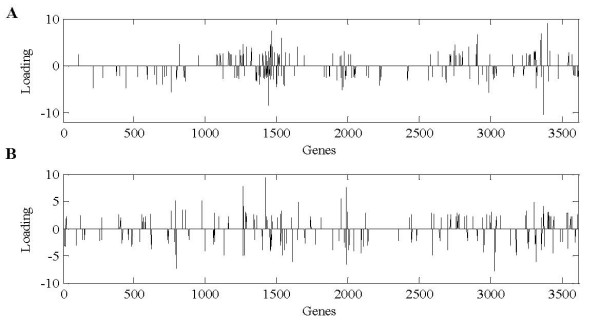
**The selected significant genes for 4-th (A) and 5-th (B) gene signatures**. Here, the threshold = 2. For reconstructing the gene expression profile, all the items whose absolute values in matrix ***S ***were less than this threshold were set to zero.

Genes with loading that exceed the chosen threshold (red line) were considered significant. Here, the threshold = 2 was used for reconstructing the gene expression profile. All of the items whose absolute values in matrix ***S ***were less than this threshold were set to zero. By testing multiple times, we achieved the threshold = 2 by which the reconstructed gene expression profile data can be successfully clustered with much fewer significant genes. The positive and negative loadings correspond to up- and down-regulation of expression, respectively. Figure 8 shows the selected significant genes.

Table 1 and 2 show the significant up- and down-regulated genes selected by the ICA method. To help further analysis, we display the gene names, their descriptions and the corresponding chromosomal locations.

**Table 1:** Selected genes up-regulated in severe AD

**Gene name**	**Description**	**Chromosomal location**
**Immunity-related protein**		
AMIGO2	adhesion molecule with Ig-like domain 2	chr12q13.11
BTG1	B-cell translocation gene 1, anti-proliferative	chr12q22
CD24	CD24 molecule	chr6q21
CD44	CD44 molecule (Indian blood group)	chr11p13
CDC42EP4	CDC42 effector protein (Rho GTPase binding) 4	chr17q24-q25
IFITM1	interferon-induced transmembrane protein 1 (9–27)	chr11p15.5
IFITM2	interferon-induced transmembrane protein 2 (1–8D)	chr11p15.5
IRF7	interferon regulatory factor 7	chr11p15.5
IFI44L	interferon-induced protein 44-like	chr1p31.1
IL4R	interleukin 4 receptor	chr16p12.1-p11.2
IRAK1	interleukin-1 receptor-associated kinase 1	chrXq28
NFKBIA	nuclear factor of kappa light polypeptide gene enhancer in B-cells inhibitor, alpha	chr14q13
		
**Metal-related protein**		
CAMK2B	calcium/calmodulin-dependent protein kinase (CaM kinase) II beta	chr22q12|7p14.3-p14.1
CALM1	calmodulin 1 (phosphorylase kinase, delta)	chr14q24-q31
CAPZA2	capping protein (actin filament) muscle Z-line, alpha 2	chr7q31.2-q31.3
CHGB	chromogranin B (secretogranin 1)	chr20pter-p12
LOC728320/LTF	lactotransferrin/similar to lactotransferrin	chr3q21-q23
MPPE1	metallophosphoesterase 1	chr18p11.21
MT1F	metallothionein 1F	chr16q13
MT1M	metallothionein 1M	chr16q13
MBP	myelin basic protein	chr18q23
SCGN	secretagogin, EF-hand calcium binding protein	chr6p22.3-p22.1
SLC24A3	solute carrier family 24(sodium/potassium/calcium exchanger), member 3	chr20p13
SLC7A11	solute carrier family 7, (cationic amino acid transporter, y+ system) member 11	chr4q28-q32
ZIC1	zinc family member 1 (odd-paired homolog, Drosophila)	chr3q24
ZBTB20	zinc finger and BTB domain containing 20	chr3q13.2
ZNF500	zinc finger protein 500	chr16p13.3
ZNF580	zinc finger protein 580	chr19q13.42
ZNF652	zinc finger protein 652	chr17q21.32
ZNF710	zinc finger protein 710	chr15q26.1
		
**Neuropeptide**		
NMB	neuromedin B	chr15q22-qter
		
**Ribosomal Protein**		
LOC644166/LO C644191/LOC728937/RPS26	ribosomal protein S26/similar to 40S ribosomal protein S26	chr12q13/chr17q21.31/chr2q31.1/chr4q26
SORBS3	sorbin and SH3 domain containing 3	chr8p21.3
		
**Cytoskeleton Protein**		
COL21A1	collagen, type XXI, alpha 1	chr6p12.3-p11.2|6p12.3-p11.2
CTBP1	C-terminal binding protein 1	chr4p16
CAPZA2	capping protein (actin filament) muscle Z-line, alpha 2	chr7q31.2-q31.3
FLNA	filamin A, alpha (actin binding protein 280)	chrXq28
		
**Cholesterol metabolism**		
APOC2/APOC4	apolipoprotein C-II/apolipoprotein C-IV	chr19q13.2
APOE	apolipoprotein E	chr19q13.2
ABCA1	ATP-binding cassette, sub-family A (ABC1), member 1	chr9q31.1
		
**Lipoprotein**		
GAD2	glutamate decarboxylase 2 (pancreatic islets and brain, 65 kDa)	chr10p11.23
LDLRAP1	low density lipoprotein receptor adaptor protein 1	chr1p36-p35
		
**Binding protein**		
AEBP1	AE binding protein 1	chr7p13
TAP1	transporter 1, ATP-binding cassette, sub-family B (MDR/TAP)	chr6p21.3
UBAP2L	ubiquitin associated protein 2-like	chr1q21.3
		
**Membrane Protein**		
HLA-DRB4	major histocompatibility complex, class II, DR beta 4	chr6p21.3
TRHDE	thyrotropin-releasing hormone degrading enzyme	chr12q15-q21
TMEM92	Transmembrane protein 92	chr17q21.33
SERPINA3	serpin peptidase inhibitor, clade A (alpha-1 antiproteinase, antitrypsin), member 3	chr14q32.1
		
**Others**		
CDKN1C	cyclin-dependent kinase inhibitor 1C (p57, Kip2)	chr11p15.5
GSTM5	glutathione S-transferase M5	chr1p13.3
SPARC	secreted protein, acidic, cysteine-rich (osteonectin)	chr5q31.3-q32

**Table 2:** Selected genes down-regulated in severe AD

**Gene name**	**Description**	**Chromosomal location**
**Immunity**		
CD22/MAG	CD22 molecule/myelin associated glycoprotein	chr19q13.1
		
**Metal-related protein**		
CABP1	calcium-binding protein 1	chr12q24.31
CACNG3	calcium channel, voltage-dependent, gamma subunit 3	chr16p12-p13.1
CAMK2B	calcium/calmodulin-dependent protein kinase (CaM kinase) II beta	chr22q12|7p14.3-p14.1
CAMK1G	calcium/calmodulin-dependent protein kinase IG	chr1q32-q41
CAPZB	capping protein (actin filament) muscle Z-line, beta	chr1p36.1
MET	met proto-oncogene (hepatocyte growth factor receptor)	chr7q31
ZNF365	zinc finger protein 365	chr10q21.2
TFRC	transferrin receptor (p90, CD71)	chr3q29
		
**Neuropeptide**		
APLP2	amyloid beta (A4) precursor-like protein 2	chr11q23-q25|11 q24
CYP26B1	cytochrome P450, family 26, subfamily B, polypeptide 1	chr2p13.3
NEFH	neurofilament, heavy polypeptide 200 kDa	chr22q12.2
NEFL	neurofilament, light polypeptide 68 kDa	chr8p21
NPY	neuropeptide Y	chr7p15.1
NTRK2	neurotrophic tyrosine kinase, receptor, type 2	chr9q22.1
SERPINI1	serpin peptidase inhibitor, clade I (neuroserpin), member 1	chr3q26.1
OLIG2	oligodendrocyte lineage transcription factor 2	chr21q22.11
NRSN2	neurensin 2	chr20p13
		
**Ribosomal Protein**		
CSPG5	chondroitin sulfate proteoglycan 5 (neuroglycan C)	chr3p21.3
C1orf115	chromosome 1 open reading frame 115	chr1q41
C20orf149	chromosome 20 open reading frame 149	chr20q13.33
C9orf16	chromosome 9 open reading frame 16	chr9q34.1
HNRPA3/HNRPA3P1	heterogeneous nuclear ribonucleoprotein A3 pseudogene 1/heterogeneous nuclear ribonucleoprotein A3	chr10q11.21/chr2q31.2
		
**Cytoskeleton Protein**		
ACTB	actin, beta	chr7p15-p12
SMARCA4	SWI/SNF related, matrix associated, actin dependent regulator of chromatin, subfamily a, member 4	chr19p13.2
		
**Oncogene**		
ABCA2	ATP-binding cassette, sub-family A (ABC1), member 2	chr9q34
ATP6V0C	ATPase, H+ transporting, lysosomal 16 kDa, V0 subunit c	chr16p13.3
ATP13A2	ATPase type 13A2	chr1p36
BCAS1	breast carcinoma amplified sequence 1	chr20q13.2-q13. 3
		
**Binding Protein**		
CABP1	calcium-binding protein 1	chr12q24.31
		
**Membrane Protein**		
RIMS3	regulating synaptic membrane exocytosis 3	chr1pter-p22.2
PCSK1	proprotein convertase subtilisin/kexin type 1	chr5q15-q21
RIMS2	regulating synaptic membrane exocytosis 2	chr8q22.3
		
**Lipoprotein**		
GRIN1	glutamate receptor, ionotropic, N-methyl D-aspartate 1	chr9q34.3
MBP	myelin basic protein	chr18q23
MOBP	myelin-associated oligodendrocyte basic protein	chr3p22.1
		
**Phosphorylation-related Protein**		
PIP3-E	phosphoinositide-binding protein PIP3-E	chr6q25.2
PLD3	phospholipase D family, member 3	chr19q13.2
PTPRT	protein tyrosine phosphatase, receptor type, T	chr20q12-q13
		
**Others**		
EIF5A	eukaryotic translation initiation factor 5A	chr17p13-p12
ISG15	ISG15 ubiquitin-like modifier	chr1p36.33
RCAN2	regulator of calcineurin 2	chr6p12.3
RGS4	regulator of G-protein signaling 4	chr1q23.3
SRD5A1	steroid-5-alpha-reductase, alpha polypeptide 1 (3-oxo-5 alpha-steroid delta 4-dehydrogenase alpha 1)	chr5p15

## Discussion

### Significant genes found by ICA

Even though the immune system tends to work less effectively in older adults than in younger ones, the elderly are prone to neuroinflammation. In fact, even though recent studies have indicated that certain aspects of the inflammatory response may have therapeutic potential [22-24], neuroinflammation is commonly believed to be a culprit in AD pathogenesis. Associated with this robust inflammatory response is the extracellular deposition of amyloid β-protein (Aβ) [25] that together are the characteristic pathological features of AD. are To validate the strong link between neuroinflammation and AD, we found that many inflammation-related genes are highly expressed, such as AMIGO2, BTG1, CD24, CD44, CDC42EP4, IFITM1, IFITM2, IRF7, FI44L, IL4R, IRAK1, NFKBIA, as Table 1 shows.

B-cell translocation gene 1 (BTG1) is a member of the anti-proliferative gene family that regulates cell growth and differentiation. Anti-proliferative, BTG1 may participate in the activation-induced cell death of microglia by lowering the threshold for apoptosis; BTG1 increases the sensitivity of microglia to the apoptogenic action of the autocrine cytotoxic mediator [26].

CD24 is a cell adhesion molecule and a cell surface glycoprotein that is expressed on both immune cells and the cells of the CNS. Literature showed that CD24 is required for the induction of experimental autoimmune encephalomyelitis (EAE), an experimental model for the human disease multiple sclerosis (MS). The development of EAE requires CD24 expression on both T cells and non-T host cells in the CNS [27].

CD44 is a multifunctional cell surface glycoprotein that serves as a receptor for hyaluronic acid, collagen types I and VI, and mucosal vascular addressin. The localization of CD44 was investigated immunohistochemically in postmortem human brain tissue of control subjects and patients with AD. Morphological diversities of CD44 positive astrocytes were in the cerebral cortex of normal subjects and patients with AD. In the AD brain, the number of CD44 positive astrocytes increased dramatically. Therefore, CD44 may be an important adhesion molecule for these astrocytic processes [28,29].

CD22 (in Table 2) is a regulatory molecule that prevents the over-activation of the immune system and the development of autoimmune diseases. Our results exhibited that CD22 is a down-expression that suggests overinflammation in AD.

Rho GTPases (Cdc42) are one of the targets in Aβ-induced neurodegeneration in AD pathology; they have a role in mediating changes in the actin cytoskeletal dynamics. The Rho family of small GTPases (Rho, Rac and Cdc42) are regulators of F-actin polymerization [30], acting as molecular switches by cycling between an inactive GDP-bound state and an active GTP-bound state. Rac1 and Cdc42 promote polymerization at the leading edge, orchestrating the formation of lamellipodia and membrane ruffles [31], as well as peripheral actin microspikes and filopodia [32,33]. RhoA is an antagonist, promoting retraction of the leading edge and assembly of stress fibers [34].

Our ICA selected results exhibited NF-κB (NFKBIA) at a high expression in severe AD (see Table 1). NF-κB plays a key role in regulating the immune response to infection. Consistent with this role, incorrect regulation of NF-κB has been linked to cancer, inflammatory and autoimmune diseases, septic shock, viral infection, and improper immune development. NF-κB has also been implicated in processes of synaptic plasticity and memory. The NF-κB activation provides the potential link between inflammation and hyperplasia.

Table 1 also shows many genes related to metal protein were up-regulated in severe AD including CAMK2B, CALM1, CAPZA2, CHGB, LOC728320/LTF, MPPE1, MT1F, MT1M, SCGN, ZIC1, ZBTB20, ZNF500, ZNF580, ZNF652, ZNF710, SLC24A3 and SLC7A11. Literature showed that the level of metal ion metabolism is closely associated with AD. For example, changes in Ca^2+ ^homeostasis, as occurring after Aβ addition, may influence several physiological responses contributing to neuronal imbalance [35]. CaMKII is a holoenzyme composed of 12 monomers, primarily α and β subunits in neurons. Autophosphorylation of CaMKIIα at Thr286 is required for normal spatial memory and place-cell representation, presumably through the triggering of its calcium-independent kinase activity [36]. Ca^2+ ^influx through the N-methyl-D-aspartate (NMDA) type glutamate receptor leads to activation and postsynaptic accumulation of Ca^2+^/calmodulin-dependent protein kinase II. NR1 and NR2B subunits of the NMDA receptor serve as high-affinity Ca^2+^/calmodulin-dependent protein kinase II docking sites in dendritic spines on autophosphorylation of Ca^2+^/calmodulin-dependent protein kinase II. Research [37,38] showed a reduction of NR1 and phosphorylated Ca^2+^/calmodulin-dependent protein kinase II levels in the frontal cortex and hippocampus of AD brains. On the other hand, Ca^2+ ^conveyed proteins CABP1, CACNG3, CAMK2B, CAMK1G, CAPZB (in Table 2) that were at low expressions in severe AD. Some primary neuron-specific transcriptional regulators that may be involved in mediating early neural development are also zinc finger-based.

Brown et al. found the level of neurofilament gene expression seems to directly control axonal diameter that in turn controls how fast electrical signals travel down the axon [39]. Our ICA selected genes (in Table 2): APLP2, CYP26B1, NEFH, NPY, NTRK2, SERPINI1, OLIG2 and NRSN2, showed that the neurofilament family is low in expression in severe AD symptoms presenting at the clinic.

To maintain cellular homeostasis, all cells must continually synthesize new proteins. Ribosomes (polyribosomes) are specialized complexes composed of nucleic acids and proteins that are responsible for mediating all protein synthesis. Impairments in protein synthesis occur in the earliest stages of AD. They occur in affected cortical regions but not the cerebellum, with impairments in protein synthesis apparently mediated by both alterations in ribosomal nucleic acids as well as the polyribosomal complex itself that suggests a novel role for alterations in protein synthesis as a potential mediator of AD pathogenesis [40]. See Table 2, the ribosomal protein: CSPG5, C1orf115, C20orf149, C9orf16, and HNRPA2/HNRPA3P1 are down-regulated in severe AD.

The changes of the cytoskeleton protein expression leads to the formation of disease, with actin filament-based structures being identified as important players in the complex pathology of AD and related dementias. A direct interaction between Tau and actin has been shown in [41,42]; actin may be a critical mediator of Tau-induced neurotoxicity in AD and related disorders. These kinds of abnormalities also showed in our ICA results for cytoskeleton protein. Some genes like COL21A1, CTBP1, CAPZA2 and FLNA were up-regulated (Table 1), whereas some genes like ACTB and SMARCA4 were down-regulated (Table 2).

APOE, which has three alleles: APOE ε2, APOE ε3 and APOE ε4, is a protein that helps to carry cholesterol and fat in the blood. APOE ε4 is regarded as the best known genetic risk factor for late-onset sporadic AD [43-47]. Aberrant cholesterol metabolism has been implicated in AD and other neurological disorders. Oxysterols and other cholesterol oxidation products are effective ligands of liver X activated receptor (LXR) nuclear receptors and major regulators of genes subserving cholesterol homeostasis. LXR receptors act as molecular sensors of cellular cholesterol concentrations and effectors of tissue cholesterol reduction. Following their interaction with oxysterols, activation of LXRs induce the expression of ATP-binding cassette, sub-family A member 1, and a pivotal modulator of cholesterol efflux. The relative solubility of oxysterols facilitate lipid flux among brain compartments and egress across the blood-brain barrier [48]. The high expression levels of APOC2/APOC4, APOE and ABCA1 can be seen in Table 1.

In addition, ICA also found some significant genes of lipoprotein, binding protein, and membrane protein etc. were up-regulated in severe AD (Table 1), such as: GAD2, LDLRAP1, AEBP1, TAP1, UBAP2L, HLA-DRB4, TRHDE, TMEM92, SPARC and SERPINA3; and some significant genes were down-regulated, such as: CABP1, RIMS3, PCSK1, RIMS2, GRIN1, MBP, MOBP, PIP3-E, PLD3, PTPRT, EIF5A, ISG15, RCAN2, RGS4, SRD5A1 (Table 2). Especially, some oncogenes like ABCA2, ATP6V0C, ATP13A2, BCAS1 had low expression levels in severe AD (Table 2).

### Significant genes found by PCA

To compare PCA with ICA, the PCA method for finding differentially expressed genes proposed by Jonnalagadda in 2008 [49] was performed on the same AD microarray data. Firstly, we modeled the control microarray data (where the samples are the variables and the gene expression measurements are the observations) using PCA and represented the expression profile of each gene as a linear combination of the dominant principal components (PCs). Then, the severe AD microarray data were projected onto the developed PCA model, and the scores were extracted. The first 100 most varied genes between the scores obtained by control data and severe AD data were selected for further biological analysis.

PCA also extracted some significant genes in immunoreactions, metal protein, membrane protein, lipoprotein, neuropeptide, cytoskeleton protein, binding protein, ribosomal protein and phosphorylation-related protein. But PCA extracted fewer genes than ICA. In immunity-related protein, PCA found only two significant genes: BCL6 and CD24 had high expression in severe AD. In metal-related protein, PCA found many up-regulated genes of the metallothionein family like: MT1P2, MT1E, MT1F, MT1G, MT1H/MT1P2, MT1X, MT2A; and CAMK2A, CALM1 and zinc finger ZBTB20, LDHA, LOC643287/PTMA. GPRC5B was the only gene found as membrane protein. In the category of lipoprotein, APOE was extracted as an important gene. For neuropeptide, NGFRAP1 and PPIA were extracted. ATP1B1 is the only phosphorylation-related protein found. Many down-regulated genes of cytoskeleton protein were extracted by the PCA method, such as: B2M, COL5A2, CSRP1, COX6A1, MAP1A, SPARC, TUBA1B, TUBA1C, TUBB, TUBB2A and TUBB2C. And PCA found many ribosomal proteins: LOC653737/LOC728501/LOC729402/LOC731567/RPL21, RPL29, RPL30, LOC342994/LOC651249/LOC729536/RPL34, RPL35, RPL4, RPL9, RPS10, RPS11, that were all down-regulated in severe AD, except one gene RPL13 that was up-regulated.

### Significant genes found by SVM-RFE

By comparing the weights of the support vectors in a sequential backward elimination manner, the Support Vector Machine Recursive Feature Elimination (SVM-RFE) method is widely used in microarray data analysis. In our experiments, to keep track of variation in gene expression associated with the development of AD, and hence, to biologically analyze the significant genes with the development of AD, the control data were treated as group 1, and the AD case data, at the first stage, were placed in group 2. With the use of SVM-RFE, by comparing the data in group 1 and group 2, the significant genes were identified; then the AD case data at the second stage (moderate) were treated as group 2, and, by comparing the gene expression data between group 1 and group 2, the significant genes were extracted. Finally group 2 consists of the AD case data at the third stage (severe), by comparing data in groups 1 and 2, the significant genes are profiled.

The SVM-RFE method found significant genes in immunoreactions such as CD44, CD74, CDC42EP4, CDK2AP1, MAL, PTMA, among which CD74 and MAL were not found by the ICA and PCA methods. Many metal metabolism-related proteins were also selected by the SVM-RFE method: MT1F, MT1H/MT1P2, MT1M, MT1X, VEZF1, ZBTB20, ZNF91, ZDHHC11, ZHX3. APOC1, USP34 and SPARC were extracted in lipoprotein, neuropeptide and secreted protein, respectively. In cytoskeleton protein, COL21A1, FGFR3, ITGB4, TPPP3, GSN, GFAP, MFAP3 were found up-regulated in severe AD. In ribosomal protein, the SVM-RFE method extracted many high expression genes like: RPL10, RPL13, RPL13A, RPL5, RPS4X, LOC387867, and two low expression genes: RNASE1 and RPL4. In the category of membrane protein, TMEM123 and LAMP2 were extracted as important genes. In phosphorylation-related protein, PIP4K2A, PDE4C, PEA15, PTPN11, PTPRK, ATP8B1, ANP32B, ABCA1, CNP were found up-regulated in severe AD. The SVM-RFE method also selected some significant oncogenes, TPT1, GLTSCR2, GUSBP1, GDF1/LASS1 that were highly expressed, as opposed to MCAM that was found to have low expression levels in severe AD.

**Figure 9:**
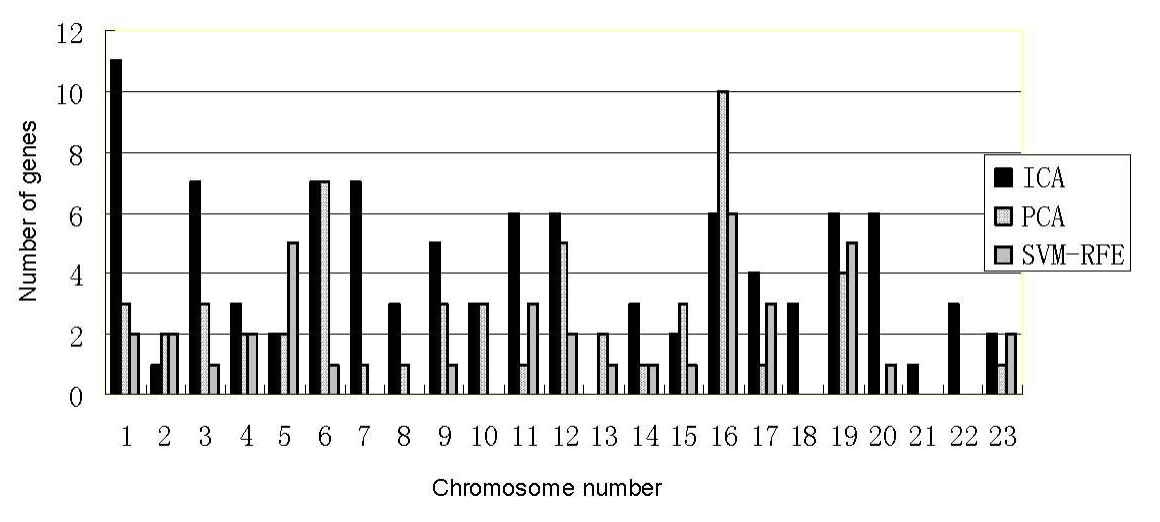
**The number of the significant genes on each chromosome selected by the ICA, PCA and SVM-RFE methods, respectively**. The ICA method extracted more significant genes on chromosome 1, 3, 4, 7, 8, 9, 11, 12, 14, 17, 18, 19, 20, 21 and 22. Especially, the genes number on chromosome 1, 3, 7 and 20 extracted by ICA were significantly higher than those obtained by PCA and SVM-RFE.

Figure 9 showed the number of the significant genes selected by the ICA, PCA and SVM-RFE methods on a different chromosome. In contrast to PCA and SVM-RFE, ICA extracted more significant genes on chromosome 1, 3, 4, 7, 8, 9, 11, 12, 14, 17, 18, 19, 20, 21 and 22. In particular, the genes numbered on chromosome 1, 3, 7 and 20, extracted by ICA, were significantly higher than ones obtained by PCA and SVM-RFE.

## Conclusion

In summary, to the best of our knowledge, this work is the first attempt to explore the power of the ICA on analyzing AD-related microarray gene expression data. By validating and identifying known and novel genes in AD-related pathogenesis, it confirms the added value of ICA over PCA and the SVM-RFE methods Our results further indicate that ICA can give researchers the ability to extract potentially disease-related genes from microarray gene expression data, and thus to delineate relevant molecular pathways of disease pathogenesis. Hence, ICA can help to elucidate the molecular taxonomy of AD and enable better experimental design to further validate and identify potential biomarkers and therapeutic targets of AD.

## Methods

Let the *n *× *m *matrix ***X ***denote the microarray gene expression data with *m *genes under *n *samples or conditions. *x*_*ij *_in ***X ***is the expression level of the *j*-th gene in the *i*-th sample. Generally speaking, the number of genes *m *is much larger than that of the samples *n*, *m*>>*n*. Suppose that the data have been preprocessed and normalized, i.e. each sample has a zero mean and standard deviation, then the ICA model for gene expression data can be expressed as:

(1) $$\left[\begin{array}{l} x_{1}(t)\\ \vdots \\ x_{n}(t) \end{array}\right]=\left[\begin{array}{ccc} a_{11} & \cdots & a_{1m}\\ \vdots & \ddots & \vdots \\ a_{n1} & \cdots & a_{nm} \end{array}\right]\left[\begin{array}{l} s_{1}(t)\\ \vdots \\ s_{m}(t) \end{array}\right]$$

And it can also be rewritten in the vector format as:

(2) ***X ***= ***AS***

In some documents, *m *× *n *matrix ***X ***was used to denote *m *genes under *n *samples. Then the transform, ***X***^T^, was used in the ICA model: ***X***^T ^= ***AS***. So, ***X***^T ^here denoted the same *n *× *m *matrix in the ICA model.

**Figure 10:**
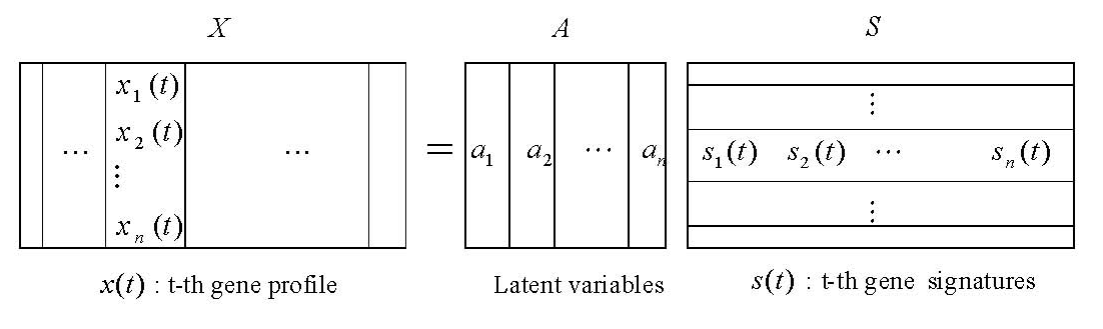
**ICA vector model of microarray gene expression data**. Matrix ***X ***denotes the microarray gene expression data with *m *genes under *n *samples or conditions. The columns of *A *= [*a*_1_, *a*_2_,..., *a*_*n*_] are the *n *× *n *latent vectors of the gene microarray data, where ***S ***denotes the *n *× *m *gene signature matrix or expression mode, in which, the rows of ***S ***are statistically independent to each other. Each gene profile provided *x*(*t*) obtained by microarray technology that was considered to be a linear combination of statistically independent components ***S ***that have specific biological interpretations and latent variables ***A***.

In the ICA model of microarray data, the columns of *A *= [*a*_1_, *a*_2_,..., *a*_*n*_] are the *n *× *n *latent vectors of the gene microarray data, ***S ***denotes the *n *× *m *gene signature matrix or expression mode, in which, the rows of ***S ***are statistically independent to each other, and the gene profiles in ***X ***are considered to be a linear mixture of statistically independent components ***S ***combined by an unknown mixing matrix ***A***. Figure 10 presents the vector framework of the ICA model.

The gene expression data provided by microarray technology is considered a linear combination of some independent components having specific biological interpretations. Lee and Batzoglou [50], and Schachtner et al. [51] gave detailed analyses for matrix ***S ***and ***A***. The *n*-th row matrix ***A ***contained the weights with which the expression levels of the *m *genes contribute to the *n*-th observed expression profile. Hence the assignment for the observed expression profiles with different classes is valid for the rows of ***A***. Each column of ***A ***can be associated with one specific expression mode. For an example of two classes, suppose one of the independent expression modes *s*_*n *_is characteristic of a putative cellular gene regulation process. It should contribute substantially to one of the class experiments whereas its contribution to another class experiments should be less, or vice versa. Since the *n*-th column of ***A ***contains the weights with which *s*_*n *_contributes to all observations, this column should show large or small entries according to the class labels. After such characteristically latent variables have been obtained, the corresponding elementary modes can be identified to yield useful information for classification. Also, the distribution of gene expression levels generally features a small number of significantly overexpressed or underexpressed genes that form very biologically coherent groups and may be interpreted in terms of regulatory pathways [3-5,10,51].

To obtain ***S ***and ***A***, the demixing model can be expressed as

(3) ***Y ***= ***WX***

**Figure 11:**
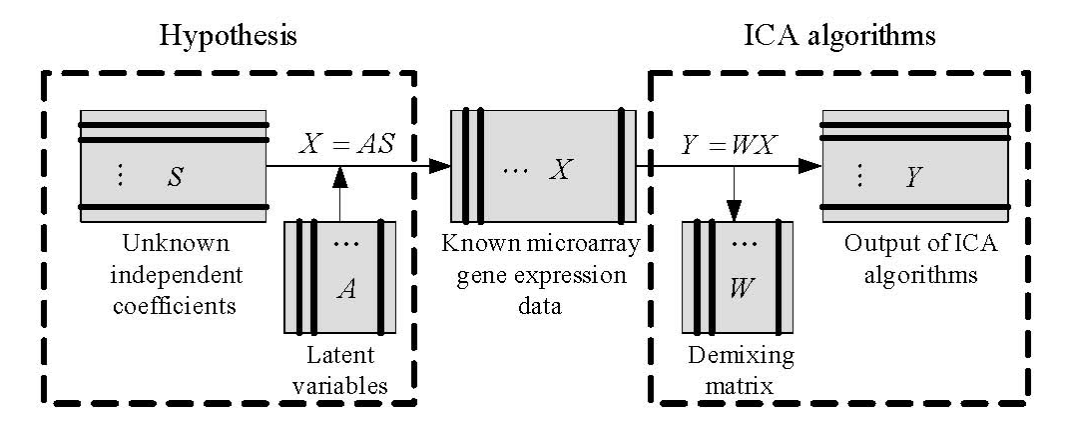
**Theoretical framework of ICA algorithms on microarray gene expression data**. In the ICA model for microarray gene expression data, the matrix ***X ***is the only one we know. According to the hypothesis (left frame) that gene profiles are linear combinations of statistically independent components ***S ***and the latent variables ***A***, the demixing process of ICA (right frame) can be applied to extract the latent variables (once we get the demixing matrix ***W***, we can obtain latent variables ***A ***easily by ***A ***= ***W***^-1^) and gene signatures that have specific biological interpretations (***Y ***is the estimator of ***S***).

Where ***W ***is an *n *× *n *demixing matrix. Figure 11 shows the processing of ICA algorithms on microarray gene expression data.

## Competing interests

The authors declare that they have no competing interests.

## Authors' contributions

WK carried out the ICA studies on the Alzheimer's DNA microarray gene expression data and drafted the manuscript. XM help with data interpretation and manuscript drafting. QL performed the data analysis using SVM-RFE method. ZC performed the statistical analysis of the data. CV and JR participated in the final data analysis and interpretation. XH conceived of the study, and participated in its design and coordination. All authors read and approved the final manuscript.
